# Cancer patients’ wish for psychological support during outpatient radiation therapy: Findings from a psychooncological monitoring program in clinical routine

**DOI:** 10.1007/s00066-018-1288-0

**Published:** 2018-03-12

**Authors:** D. Riedl, R. Gastl, E. Gamper, C. R. Arnold, D. Dejaco, F. Schoellmann, G. Rumpold

**Affiliations:** 1 University Clinic of Medical Psychology, Medical University of Innsbruck, Innsbruck, Austria; 2 Innsbruck Institute of Patient-reported Outcome Research (IIPCOR), Innsbruck, Austria; 3 Department of Therapeutic Radiology and Oncology, Innsbruck Medical University, Innsbruck, Austria; 4 Department of Otorhinolaryngology, Medical University of Innsbruck, Innsbruck, Austria

**Keywords:** Psychooncology, Distress screening, Treatment wish, Prevalence, Treatment path, Psychoonkologie, Belastungsscreening, Behandlungswunsch, Prävalenz, Behandlungspfade

## Abstract

**Background:**

Cancer patients frequently suffer from physical and psychosocial impairments due to their disease and its treatment. Psychooncology (PO) can help to cope with stress resulting from outpatient radiotherapy (RT) treatment. There are currently few data regarding patients’ wishes for PO support. The aim of this study was to investigate the number of patients with a wish for PO, treatment paths, and predictors of the wish for PO among cancer patients at the beginning of RT.

**Methods:**

The results of routine psychological stress screening (Hornheide screening instrument; cut-off  ≥ 4) of 944 cancer patients between 2015 and 2017 were analyzed in a retrospective cross-sectional study. Predictors for a wish for PO support were identified by stepwise binary logistic regression, in which sociodemographic and treatment data were included in addition to the screening items.

**Results:**

Around 20% of patients had above-average stress levels and 13% expressed a wish for PO support (participation rate was approximately 55%). Low emotional wellbeing (OR = 11.3) and lack of social support (OR = 9.4) were strong predictors for this treatment wish. Among patients with pancreatic cancer, head and neck tumors, and hematologic disease, there was a substantial difference between the degree of psychological stress and the wish for treatment. Patients with urological (23.5%) and lung tumors (20.9%) most frequently expressed a wish for PO support.

**Conclusion:**

Patient-reported psychosocial problems were better predictors of a wish for PO support than sociodemographic or clinical data. Stress screening should thus be implemented in clinical routine.

**Electronic supplementary material:**

The online version of this article (10.1007/s00066-018-1288-0) contains supplementary material, which is available to authorized users.

## Zusammenfassung

**Hintergrund:**

Krebspatienten leiden aufgrund der Erkrankung und Behandlung häufig unter körperlichen und psychosozialen Beeinträchtigungen. Psychoonkologie (PO) kann helfen, solche Belastungen im Zuge der ambulanten Strahlentherapie (RT) zu verarbeiten. Gegenwärtig liegen jedoch nur wenige Daten vor, inwieweit sich Patienten eine PO-Behandlung wünschen. Ziel war es, die Anzahl der Patienten mit PO-Behandlungswunsch, Behandlungspfade sowie Prädiktoren des Betreuungswunschs bei Krebspatienten zu Beginn der ambulanten RT zu untersuchen.

**Methode:**

Zwischen 2015 und 2017 wurden die Ergebnisse eines routinemäßigen psychischen Belastungsscreenings (Hornheide-Screening-Instrument; Cut-Off ≥ 4) von 944 Krebspatienten in einer retrospektiven Querschnittsanalyse untersucht. Prädiktoren für den PO-Behandlungswunsch wurden mittels schrittweiser binärer logistischer Regression identifiziert. Dabei wurden soziodemographische und behandlungsbezogene Daten sowie Screening-Items einbezogen.

**Ergebnisse:**

Etwa 20 % der Patienten zeigten überdurchschnittliche Belastungswerte und 13 % wünschten sich eine PO-Behandlung (Partizipationsrate ca. 55 %). Geringes emotionales Wohlbefinden (OR = 11,3) und fehlende soziale Unterstützung (OR = 9,4) waren starke Prädiktoren für den Behandlungswunsch. Zwischen dem psychischen Belastungsgrad und dem Betreuungswunsch zeigte sich bei Patienten mit Pankreaskrebs, Hals-Kopf-Tumoren sowie hämatoonkologischen Erkrankungen ein substantieller Unterschied. Am häufigsten wünschten sich Patienten mit Krebs in den Harnorganen (23,5 %) und Lungenkrebs (20,9 %) eine PO-Behandlung.

**Schlussfolgerung:**

Patientenberichtete psychosoziale Probleme waren bessere Prädiktoren für den Wunsch nach PO-Behandlung als soziodemographische oder klinische Daten. Belastungsscreenings sollten daher in die klinische Routine implementiert werden.

## Background

Despite improved survival rates over the past two decades due to diagnostic and therapeutic advances, cancer remains the second leading cause of death worldwide [1]. A substantial number of cancer patients report short- and long-term psychological distress due to the disease, active treatment, and treatment consequences [2, 3]. Between 22% and 35% of radiotherapy outpatients report clinically relevant psychological distress [4–7]. High levels of psychological distress warrant clinical attention, since they may negatively influence treatment adherence [8, 9], satisfaction with care [10], and health-related quality of life (QoL; [11]). Available data suggest that psychooncological support (PO) can effectively reduce psychological distress and improve patients’ QoL [12–14]. However, a large proportion of distressed cancer patients do not receive professional support [14–16]. Multifaceted reasons for this mismatch have been discussed in the literature. Firstly, screening for psychological distress by clinicians is unsatisfactory, resulting in an underestimation of patients’ distress levels and a lower number of referrals [4, 17]. Routine distress screening can significantly increase the number of referrals to PO [18]: when actively offered during radiotherapy, 13–41% of the patients accepted referral [6, 19, 20]. Incorporation of such screening procedures into clinical routine to effectively identify distressed cancer patients to enable quick and adequate PO is recommended [21, 22]. Screening is usually conducted with short, easily applicable and interpretable questionnaires, followed up by referral to a more specialized healthcare professional if necessary [23].

Secondly, cancer patients frequently misjudge their own distress levels as not severe enough for PO [24], and restrain from self-referral when in need [25]. This phenomenon was recently described as “normality paradox” by Carolan et al. [26]. Patients aim at maintaining a feeling of normality by rejecting professional support. Additional barriers include lack of local services or financial considerations [27], lack of time or awareness of services, and patient refusal [25]. Higher pain levels, increased support requirement in daily life, increased patient-reported symptoms, and decreased functional status were previously observed as influential factors on self-referral rates to PO. In contrast, clinical characteristics (i.e., tumor entity, presence of metastases) and sociodemographic factors were reported to be non-predictive [6].

In short, available data suggest that cancer patients significantly benefit from PO, but clinicians and patients themselves restrain from (self-) referral for various reasons. Besides incorporating a more active pattern of referral to available services in clinical practice, factors influencing a patient’s decision towards self-referral should be explored to identify patients at risk. However, such data collected from large mixed samples during routine assessment, rather than in controlled experimental environments, are rare.

Here, we present data from a routine psychooncological screening procedure during outpatient radiotherapy. In our study, we addressed the following aims: (a) to assess the number of patients who wish for PO across tumor entities, (b) to identify predictors for the wish for PO, and (c) to describe the referral pathways for PO in clinical routine.

## Methods

We retrospectively analyzed data collected in clinical routine from cancer outpatients treated at the Department of Therapeutic Radiology and Oncology (Medical University of Innsbruck) between January 2015 and January 2017.

Patients were included if they were (a) treated for cancer, (b) outpatients, and (c) older than 18 years.

### Clinical procedure

At the Department of Therapeutic Radiology and Oncology, approximately 160–180 patients are treated daily; the vast majority are outpatients. Routine treatment includes the possibility of PO, social work, nutrition counseling, or creative therapy.

The treatment staff (i.e., physicians, radiation therapists, nurses, social workers) offer referral to PO if cancer patients seem distressed. Additionally, all cancer outpatients are asked to complete an eight-item screening questionnaire regarding their need for PO, which is handed out to the cancer patients within the first 3 days of radiotherapy by the staff, and can be returned to the treating physicians or other staff at the radiation units. Participation in the screening procedure is voluntarily and non-participation results in no disadvantage to the cancer patients. The leading psychologist collects the screening tests twice a week, and patients who either had a score above the cut-off and/or wished for PO are contacted soon after.

**Fig. 1:**
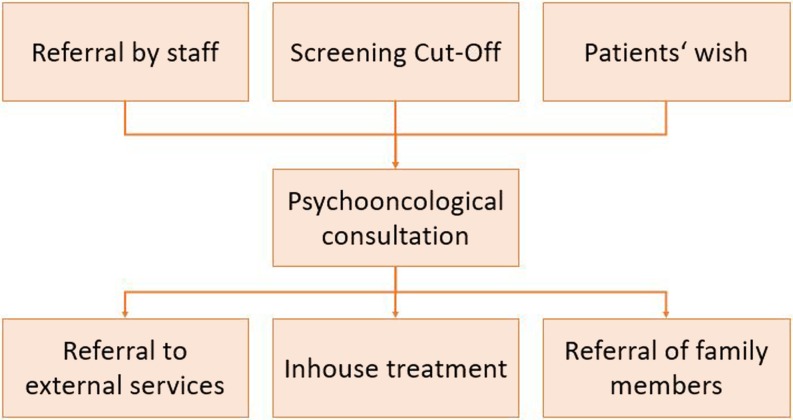
Treatment flow

Depending on the patients’ needs, they receive PO throughout radiotherapy or are referred to external psychosocial services. Referral may include cost-free PO counseling centers close to home, outpatient psychiatrist, specialized psychotherapists, or counseling by social workers. Furthermore, support is offered to distressed family members. This often comprises providing information or referring to external services (see Fig. 1).

### Screening questionnaire and data collection

To assess the need for PO, the Hornheide Screening Instrument (HSI) was used. The HSI consists of seven items assessing physical and emotional well-being, additional emotional burden unrelated to disease, social support, burden on family through hospital stay, the inability to calm down, and the level of information about disease and treatment. The items can be added up to a total score (range 0–14). Values ≥ 4 indicate the *need* for PO.

We added one further dichotomous item asking patients if they *wish* for PO.

### Statistical analysis

Descriptive statistics were calculated to describe the sample, prevalence of the wish for PO, and referral pathways for PO. To identify factors that predicted the *wish* for PO as indicated by the dichotomous variable, we conducted a stepwise binary logistic regression. In a first step, we tested the association of sociodemographic and clinical variables (cancer sites, previous treatments, histological grading, disease state, and treatment concept) and in a second step the association of the seven HSI items. Significant predictors of each block were selected using the backward elimination method (likelihood ratio test) and were entered in the final model. Educational levels were dichotomized into low (below higher school certificate) and high (at least higher school certificate) education. We included the three most frequent tumor locations (breast, prostate, and lung) as categorical variables and consolidated the remaining tumor locations into a single group. Odds ratios (OR) are presented with 95% confidence intervals (CI).

## Results

Between January 2015 and January 2017, approximately 1700 cancer outpatients were treated at the Department of Therapeutic Radiology and Oncology. A total number of 961 cancer patients returned the screening instrument, leading to a participation rate of approximately 55%. Of these, 17 cancer patients (1.8%) were excluded since they did not meet the inclusion criteria. Five cancer patients did not complete the screening questionnaire, but stated their wish for PO and were therefore kept in the analyses. For the finally included 944 cancer patients, mean age was 63.9 years, 50.6% were female, 68.9% were married or in a long-term relationship, and 85.0% had children. About half of the cancer patients had finished compulsory school and an apprenticeship (55.5%), and almost a third (30.0%) had higher education or a university degree.

The most frequent tumor locations in the cohort were breast, prostate, and lung cancer. The proportion of patients with breast cancer was comparable to the total population of outpatients treated at the department (33.9% vs. 31.4%), while patients with prostate cancer were slightly overrepresented (22.1% vs. 18.8%) and patients with lung cancer slightly underrepresented (12.3% vs. 14.9%).

Most cancer patients (73.0%) were treated for a primary disease and with a curative treatment plan. Within the three most frequent tumor locations, patients with breast cancer and prostate cancer were mostly treated with a curative treatment plan (91.9% and 89.4%, respectively), while palliative treatment was more frequent in patients with lung cancer (47.8%). The majority of the cancer patients (89.6%) had a Karnofsky Performance Score between 80 and 100. For details on clinical data, see Table 1.

**Table 1:** Clinical data

	*n*	%
*Cancer * *sites* ^a^		
Breast cancer (C50)	320	33.9
Prostate cancer (C60-63)	209	22.1
Lung cancer (C33-34)	116	12.3
Head and neck cancer (C00-14; C30-33)	49	5.2
Colorectal cancer (C18-21)	49	5.2
Brain cancer (C70-72)	40	4.2
Hemato-oncological cancer (C81-96)	40	4.2
Malignancy of connective and soft tissue	21	2.2
Gynecological tumors (C51-58)	19	2.0
Melanoma (C43-44)	19	2.0
Urinary organs (C64-68)	16	1.7
Secondary and ill-defined (C76-80)	15	1.6
Gastric cancer (C15-17)	11	1.2
Pancreatic cancer (incl. liver and gall bladder: C22-25)	10	1.1
Other	10	1.1
*Previous treatments*		
Radiotherapy	75	7.9
Surgery	422	44.6
Chemotherapy	143	15.1
Hormonal therapy	232	24.5
Immunotherapy	27	2.9
*Histological grading*		
Grade I	58	6.1
Grade II	422	44.7
Grade III	226	23.9
Grade IV	36	3.8
Missing values	202	21.4
*Treatment concept*		
Curative	748	79.2
Palliative^b^	196	20.8

^a^ICD-10 codes

^b^Palliative treatment medical treatment of incurable diseases aiming primarily at symptom control and improvement of quality of life

### Prevalence of the wish for psychooncological support

Most cancer patients (>90%) reported medium or good physical and emotional well-being and having someone to talk to about concerns and fears. Yet, about 17.2% reported that their illness affected family members and that they were currently troubled by other topics. The vast majority of cancer patients felt well informed about their disease and treatment.

**Fig. 2:**
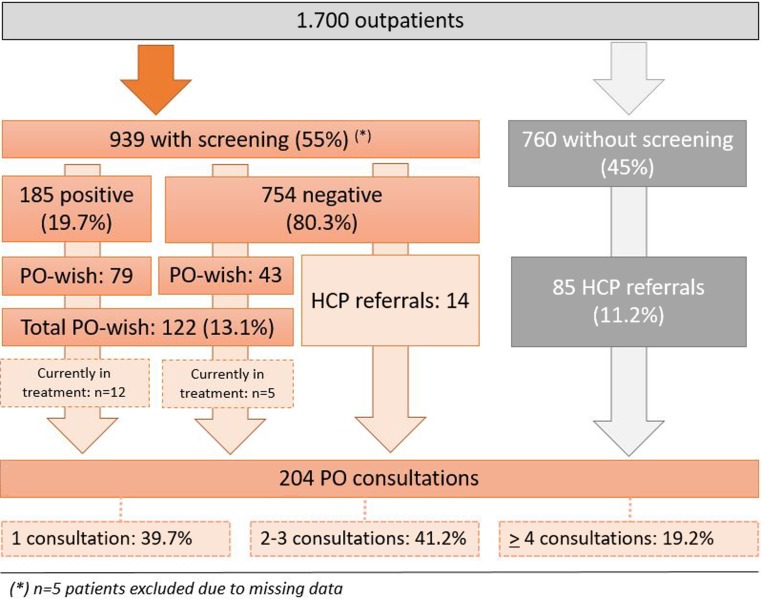
Description of the referral pathways for patients with (*left side*) and without screening (*right side*). *PO* psychooncological support, *HCP* health-care professional

In total, 185 cancer patients (19.7%) were identified as potentially in need of PO (HSI score ≥ 4), and 42.7% of these wished for PO. Another 5.7% of the cancer patients below the cut-off also wished for PO, resulting in a total of 13.1% of all included cancer patients. Some of these cancer patients already received PO and were therefore not contacted by a psychooncologists. Healthcare professionals referred 14 cancer patients with negative screening results who had not stated a wish for PO and another 85 cancer patients who had not returned their questionnaire. This resulted in 204 cancer outpatients who received PO during their radiotherapy. Of these patients, 39.7% received one consultation, 41.2% received 2–3 consultations, and the remaining 19.2% received 4–9 consultations. For details see Fig. 2.

A large proportion (40.7%) of the cancer patients who were consulted only once were referred to other healthcare services, mostly to cost-free PO counseling centers close to home (33.3%), to outpatient psychiatrists (30.3%), or to psychotherapists (21.2%). Another 12.1% of these cancer patients were referred to the inhouse social worker for social counseling; 6.4% aborted the PO. Cancer patients who had not taken part in the screening procedure were referred to external services significantly more often than patients with available screening data (63.6% vs. 36.4%; Chi^2^ = 6.2, *p* = 0.013).

**Fig. 3:**
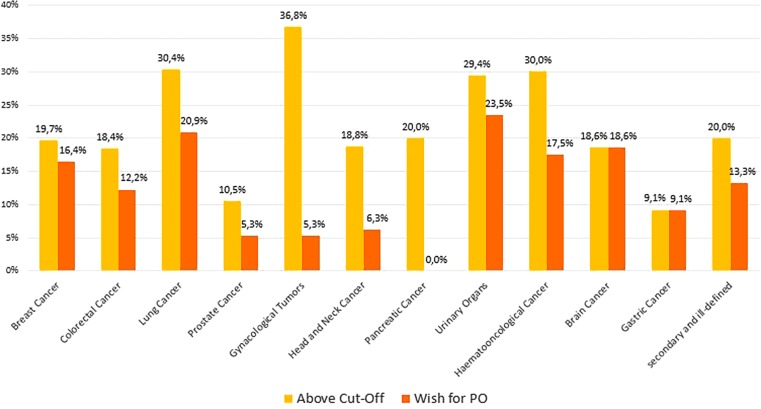
Percentage of patients above the HSI cut-off and percentage of patients who wish for psychooncological support

The number of cancer patients above the cut-off strongly varied amongst cancer types, showing the highest percentage amongst patients with gynecological cancer, cancer in the urinary organs, and hemato-oncological malignancies. The lowest proportion of distressed patients was found in gastric cancer and prostate cancer. As shown in Fig. 3, there was some variation between the percentage of patients above the proposed cut-off and the number of patients who wished for PO amongst some cancer entities. While the HSI cut-off seemed to reflect the wish for PO well in patients with brain cancer, gastric cancer, and breast cancer, our results showed large differences in patients with gynecological tumors, pancreatic tumors, and head and neck cancer.

### Association of sociodemographic and clinical characteristics with the subjective need for psychological support

The omnibus test showed that the logistic regression model was statistically significant (χ^2^ = 209165, *p* < 0.001) and explained 42.5% (Nagelkerke R2) of the variance. The model was able to correctly classify 90.5% of the cases, which indicates excellent adequacy.

All nine variables entered in the final model remained statistically significant. Highest odds ratios regarding the wish for PO were found in patients with low emotional well-being (OR = 11.3) and lack of social support (OR = 9.4). The significant predictors included lower age, higher level of education, lower emotional well-being, additional emotional burden unrelated to disease, less social support, burden on family through hospital stay, and the inability to relax. Regarding the type of cancer, there was an overall association of diagnosis with the dependent variable: patients with lung cancer had 2.3-times and patients with breast cancer 1.9-times higher odds to wish for PO compared to the combined remaining diagnoses (see Table 2).

**Table 2:** Multivariate logistic regression model: Influential factors on patients wish for psychooncological support

	Regression coefficient B	Wald	df	Sig	Adjusted OR	95% confidence interval for OR	
						Lower	Upper
Age	−0.02	4.59	1	0.032	0.98	0.96	0.99
Level of education (dichotomized; high vs. low education)	0.73	6.95	1	0.008	2.07	1.21	3.55
Diagnosis	–	8.80	3	0.032	–	–	–
*Diagnosis: others* ^*a*^	*0.00*	*–*	*–*	*–*	*1.0*	*–*	*–*
*Diagnosis: breast cancer*	*0.65*	*3.79*	*1*	*0.051*	*1.91*	*0.99*	*3.66*
*Diagnosis: prostate cancer*	*−0.29*	*0.37*	*1*	*0.546*	*0.75*	*0.29*	*1.92*
*Diagnosis: lung cancer*	*0.82*	*4.27*	*1*	*0.039*	*2.28*	*1.04*	*4.97*
HSI Item 2: emotional well-being	–	32.10	2	<0.001	–	–	–
*HSI Item 2: rather good* ^a^	*0.00*	*–*	*–*	*–*	*1.000*	*–*	*–*
*HSI Item 2: medium*	*1.24*	*17.50*	*1*	<0.001	*3.44*	*1.93*	*6.13*
*HSI Item 2: rather bad*	*2.46*	*26.9*	*1*	<0.001	*11.69*	*4.62*	*29.59*
HSI Item 3: Additional emotional burden unrelated to disease	0.69	5.00	1	0.025	1.99	1.09	3.65
HSI Item 4: Lack of social support	2.24	28.57	1	<0.001	9.39	4.13	21.34
HSI Item 5: Burden on family through hospital stay	0.99	11.03	1	0.001	2.71	1.51	4.89
HSI Item 6: Inability to calm down	1.03	7.77	1	0.005	2.80	1.36	5.78

*OR* odds ratio, *df* degree of freedom, *HSI* hornheide screening instrument

^a^category taken as reference group

We found no significant association of relationship status, parenthood, disease status (initial manifestation, metastases, secondary tumor, tumor recurrence), previous cancer-related treatments, and two HSI items (physical well-being, level of information about disease and treatment) on the dependent variable and therefore did not include these variables in the final model.

## Discussion

PO aids patients in coping with various challenges that may occur during oncological radiotherapy. To facilitate optimal identification of distressed cancer patients, a stepwise approach was implemented at our Department. Firstly, all cancer patients are screened for distress using evaluated questionnaires. Secondly, all cancer patients who score above a predefined cut-off, articulate a need for PO, or are identified as distressed by the healthcare staff are contacted by a specialized psychologist and are then thirdly either treated at the unit or referred to appropriate external services. This approach is based on guidelines (i.e., [22]) as well as on our practical and clinical experiences at the department.

The aim of our study was to investigate how many cancer patients wished for PO during outpatient radiotherapy in this real-world setting. Our secondary goals were to investigate factors influencing the wish for PO and to describe the referral pathways for PO in clinical routine.

In the present cohort, 19.6% of the cancer patients reported values above the clinical cut-off and 13.1% of all cancer patients wished for PO. These incidence rates are in line with previous studies with a similar study design [5–7]. In controlled and experimental studies, reported incidence rates were significantly lower [19, 20]. In these studies, the assessment periods were shorter and samples were smaller within predefined cancer subtypes. This underscores the importance of real-life data to evaluate the need for PO in cancer patients.

We observed eight variables, each independently influencing the wish for PO, namely lower emotional well-being, lack of social support, burden on family through hospital stay, the inability to calm down, and higher level of education. Furthermore, younger patients and patients with lung cancer or breast cancer had a significantly higher probability to wish for PO. Our observations suggest that self-reported psychosocial issues were significantly better predictors for the wish for PO than sociodemographic or clinical variables (i.e., sex, parenthood, previous oncological treatments, disease state), which is in line with previous research [28].

In the present cohort, patients with gynecological cancer, lung cancer, and hematological malignancies reported the highest levels of psychosocial distress. Previous research indicated that cancer patients from different entities differed in their need for PO: Singer et al. [29] reported that the wish for PO varied between 15 and 45% across different cancer entities in a large sample of mixed oncological inpatients. The largest proportion of patients wishing for PO in our study was found amongst patients with cancer of the urinary tract, lung cancer, and brain cancer. Acceptable accordance was observed between the level of psychosocial distress and the wish for PO in patients with breast cancer, brain cancer, or gastric cancer.

In contrast, discrepancy between psychosocial distress and acceptance of PO for other cancer types was profound: the largest discrepancies were found for patients with gynecological cancer, pancreatic cancer, head and neck cancer, and hematological cancer. This indicates that the HSI cut-off of ≥4 may not be equally fit for all cancer entities. We therefore decided to add the Distress Thermometer to our screening procedure from now on, to gain more insight about suitable cut-off values for the different tumor entities.

Other reasons for this mismatch may be that patients with certain cancer types are more reluctant to accept PO: patients with head and neck cancer, for example, often suffer from a broad range of comorbidities [30], have a higher chance to suffer from depression [29], and are more likely to commit suicide than patients with other types of cancer [31]. Yet, in accordance with our findings, they are also less likely to ask for PO [29, 30]. In a recent study, Faller et al. [32] observed that while patients with gynecological cancer reported lower QoL than patients with breast cancer, there was no difference regarding their wish for PO. In our sample, patients with gynecological tumors most frequently reported values above the cut-off, yet the wish for PO was amongst the lowest of the whole sample. Regarding the subgroup of patients with gynecological cancer and pancreatic cancer, however, our results should be interpreted with great caution, since the samples were quite small. Nevertheless, our data suggest that further exploration of reasons for acceptance or rejection of PO (e.g., [33]) is warranted to improve clinical care for patients with all cancer entities.

In our understanding, especially in busy outpatient units, the combination of personal referral and short and quick screening procedures is a valid strategy to prevent the underdiagnoses of psychosocial distress. Interestingly, several patients wished for PO despite scoring below the proposed cut-off. This indicates that self-administered screening procedures may also encourage patients to seek PO as a preventive strategy or because of problems not related to their oncological disease. Also, cancer patients receive a feedback about their distress level, which may correct possible misjudgment of their own distress levels as not severe enough for PO.

Here, 204 cancer patients received at least one consultation. About 60% of the patients were seen more than once by a psychooncologist, with up to nine sessions per patient. Of the patients who were consulted only once, about 40% were referred to other healthcare services. This proposes that especially in treatment units with larger catchment areas, it is important to inform patients about treatment options closer to their home. Since many cancer patients face financial challenges, the integration of counseling by social workers at the treatment units is necessary.

For 40% of the cancer patients who received a psychooncological consultation, no screening data were available. This indicates two things: (a) screening can only be an additional tool to personal referral by healthcare professionals and (b) patients who do not return the screening instrument may also be distressed and may also profit from PO. No information on the reasons for non-participation in the screening procedure in our study was available. We have planned to further investigate reasons for non-participation at our center.

This topic is especially interesting to us since our data show that patients who had PO without prior screening information (i.e., referred by healthcare professionals) were consecutively referred to external services significantly more often than patients with screening results. A possible explanation could be that distressed patients who do not wish for PO at the unit may not know about the option to be referred to external services closer to home, and are therefore more reluctant to participate in the screening procedure. Nevertheless, in our experience, many of those patients still benefited from PO. The dropout rates in the paper and pencil assessment therefore hinder the optimal treatment and referral. The implementation of routine electronic screening procedures could help to minimize this effect and therefore help to better identify distressed patients.

We observed that patients are more likely to complete screening questionnaires if they experience active incorporation of the questionnaires’ results into their individual treatment plan. We experienced that including the staff at the radiation units in the screening procedure (i.e., handing out the questionnaires) aided in implementing PO in the patients’ clinical routine. This may facilitate de-stigmatization. Since cancer patients are regularly seen by the same staff during radiotherapy, they can be encouraged to accept PO help if necessary. Furthermore, routine screening might help patients to accept PO by (a) pointing out that such services exist and (b) helping patients to identify specific problems by asking specific questions.

### Limitations

One major limitation of our study was the participation rate of approximately 55%. While this is comparable to other retrospective analyses of paper and pencil data collected in clinical routine [34, 35], it still hinders the generalizability of our observations. Moreover, we do not know the reasons for non-participation or whether all patients received a screening questionnaire. One influence on the non-completion rate may be the mode of assessment: Gamper et al. [34] found higher non-completion rates and poorer adherence rates for paper and pencil routine assessments compared to electronic assessments. Electronic assessments enable the psychologists to approach the patients more quickly, since screening results are available immediately after completion. Previous studies indicated that the implementation of electronic assessments during outpatient radiotherapy is feasible, and the majority of patients are highly willing to complete electronic assessments [5, 7, 36].

Also, screening was only conducted once, at the beginning of radiotherapy. While side effects mostly do not occur at the beginning of the treatment, physical well-being is likely to decrease during the course of the treatment. Additional screening during and/or at the end of the treatment phase may result in higher incidence numbers of psychosocial distress and a higher number of patients wishing for PO. Based on the comparably high number of patients who were additionally referred to PO during the study period, our incidence numbers may even underrepresent the true number of patients who would require PO.

Finally, the retrospective study design prevents us from evaluation of the proportion of patients who might had been referred to PO even if no screening would have been performed. However, previous studies observed that oncologists tend to underestimate the patients’ distress levels and their need for PO [4, 17].

## Conclusion

PO is an important supportive therapy during radiotherapy in cancer patients, especially for emotionally distressed and socially deprived patients. To facilitate the early detection and referral of distressed patients, screening should be integrated into routine clinical care. Routine distress screening may be helpful to further de-stigmatize PO by presenting it as normal part of clinical routine. However, paper and pencil-based assessment procedures impede the immediate use of the test results and may lead to lower response rates. The electronic collection of screening questionnaires might facilitate referral and improve participation rates in the screening procedure.

## Caption Electronic Supplementary Material

Hornheide Screening Instrument (HSI)
