# Supplementary material for: Cancer patients’ wish for psychological support during outpatient radiation therapy: Findings from a psychooncological monitoring program in clinical routine
Source: Strahlenther Onkol. 2018 Mar 12;194(7):655–63. doi: 10.1007/s00066-018-1288-0 (PMC6008369; doi:10.1007/s00066-018-1288-0)
Supplement: Supplementary file 1 — Hornheide Screening Instrument (HSI) [file 66_2018_1288_MOESM1_ESM.docx]

Hornheide Screening Instrument (HSI)

|  | |  |  |
| --- | --- | --- | --- |
| 1. How did you feel physically during the last 3 days? | rather good (0) | medium (1) | rather bad (2) |
| 2. How did you feel mentally and emotionally during the last 3 days? | rather good (0) | medium (1) | rather bad (2) |
| 3. Besides the current illness, is there something else that troubles you? | yes (2) | no (0) | |
| 4. Do you have somebody to talk to about your concerns and fears? | yes (0) | no (2) | |
| 5. Does your hospital stay significantly affect other family members? | yes (2) | no (0) | |
| 6. Are you able to relax and find peace in mind during daytime? | yes (0) | no (2) | |
| 7. How well do you feel informed about the disease and your treatment? | rather good (0) | medium (1) | rather bad (2) |
| Total score | ……………………………. |  |  |
| Do you wish for psychological support? | yes  ( ) | no  ( ) | |
